# Local Administration of Bone Morphogenetic Protein-2 Using a Hydrogel Carrier for Robust Bone Regeneration in a Large Animal Model of Legg-Calvé-Perthes disease

**DOI:** 10.21203/rs.3.rs-2465423/v1

**Published:** 2023-01-17

**Authors:** Harry Kim, Chi Ma, Minsung Park, Felipe Monte, Vishal Gokani, Olumide Aruwajoye, Yinshi Ren, Xiaohua Liu

**Affiliations:** Scottish Rite for Children; Scottish Rite for Children; Scottish Rite for Children; Scottish Rite for Children; Scottish Rite for Children; Scottish Rite for Children; Scottish Rite for Children; Texas A&M School of Dentistry

## Abstract

Legg-Calvé-Perthes disease is juvenile idiopathic osteonecrosis of the femoral head (ONFH) that has no effective clinical resolutions. Previously, local injection of bone morphogenetic protein-2 (BMP2) for ONFH treatment showed a heterogeneous bone repair and a high incidence of heterotopic ossification (HO) due to the BMP2 leakage. Here, we developed a BMP2-hydrogel treatment via a transphyseal bone wash and subsequential injection of BMP2-loaded hydrogel. In vivo studies showed that a hydrogel of gelatin-heparin-tyramine retained the BMP2 for four weeks. The injection of the hydrogel can efficiently prevent leakage. With the bone wash, the injected hydrogel had a broad distribution in the head. In vivo studies on pigs revealed that the BMP2-hydrogel treatment produced a homogeneous bone regeneration without HO. It preserved the subchondral contour and restored the subchondral endochondral ossification, although it increased growth plate fusions. In summary, the study demonstrated a promising BMP2-hydrogel treatment for ONFH treatment, especially for teenagers.

## Introduction

Legg-Calvé-Perthes disease (LCPD) is a childhood ischemic osteonecrosis of the femoral head (ONFH) that affects 1 in 1200 children ^1^. It is one of the most serious conditions affecting the pediatric hip joint, especially in teenagers, as over 50% of patients will develop debilitating osteoarthritis despite receiving treatments. ^2–4^ ONFH causes the local death of osteocytes and bone marrow cells due to a disruption of blood supply to the bone, and the subsequent repair process results in necrotic bone resorption and structural deformities.^5^ The progression of LCPD leads to hip pain, limited range of motion, and physical disability necessitating a total hip replacement (THR).^6^ However, THR is not an optimal treatment option for young patients due to their high physical demand and longevity.

Current treatments to prevent the progression of LCPD, such as weight-bearing restrictions, bracing, and osteotomies show poor clinical outcomes, especially in teenagers with LCPD.^7,8^ Single-tunnel decompression or multiple epiphyseal drillings are common operative treatments to decompress necrotic bone. ^9–11^ It is believed that decompression can create intraosseous tunnels to facilitate revascularization and repair of the necrotic femoral head. Clinical studies, however, showed that decompression alone has limited treatment efficacy, and the failure rate can reach 50%.^12–14^

Bone morphogenetic protein 2 (BMP2) belongs to the transforming growth factor beta family and has strong osteogenic potential.^15–17^ Exogenous recombinant human BMP2 has been approved for tibial non-union fractures and spinal fusion.^18^ It is reported that local administration of BMP2 can significantly increase new bone formation and decrease the risk of femoral head deformation. ^19–21^ Saline has been used as a carrier for local BMP2 injection to treat ONFH. However, using saline as the carrier was associated with a high incidence of heterotopic ossification (HO) within the hip capsule ^22–24^ as it has little ability to confine the leaking of BMP2 during the injection. The leakage also reduces the local dose and distribution at the target site and results in incomplete bone regeneration and heterogeneous bone repair.^20^

Orthopaedic biomaterials, such as granular or sponge bone grafts, have been tested as BMP2 carriers for the treatment of ONFH. ^19,25^ These modalities can be compacted into drilled tunnels. With such application, osteoinduction only occurred within the region of the drilled tunnels, and not in the rest of the necrotic bone. Injectable bone cement (polymethylmethacrylate, PMMA) exhibited a broad distribution within the bone after injection by spreading into the trabecular space. It also provided high mechanical support after setting. However, the setting of PMMA generates a high local temperature which inactivates BMP2.^26^ To the best of our knowledge, there are no clinically available tissue engineering strategies that can be used as an effective treatment of ONFH.

An ideal tissue engineering strategy for the treatment of ONFH requires delivering effective bioactive factors (such as BMP2) to the necrotic head with broad distribution and provides sustained osteoinduction. Injectable hydrogels are able to adapt to different shapes in realtime and are widely used as minimally invasive cell/drug carriers in bone tissue regeneration. ^27^ In our previous study, the distribution of a pre-clinical hydrogel was tested on an *ex vivo* ONFH model, which demonstrated that the hydrogel was injected into the trabecular space beyond the drilled tunnels.^28^ Furthermore, we recently developed a bone wash technique to remove necrotic bone marrow and debris from the necrotic area.^29^ It is postulated that a local necrotic bone wash may better facilitate the delivery of injectable biomaterials. Moreover, we previously reported a heparin-modified gelatin molecule could specifically bind to bioactive proteins of the transforming growth factor beta family, involving BMP2 and VEGF. This binding effect prevented the bioactive proteins from denaturation and proteolytic degradation. ^30,31^

**Figure 1:**
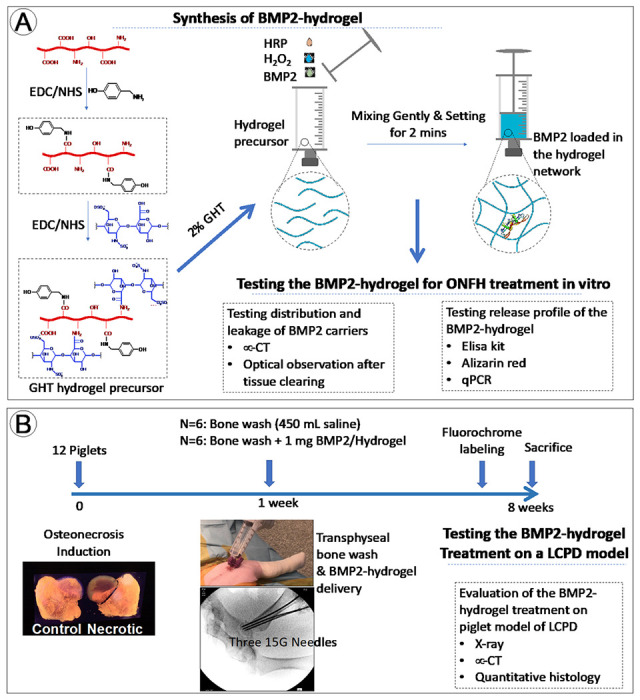
Illustration of the tissue engineering design for the treatment of LCPD. A) The synthetic route of the gelatin-heparin-tyramine hydrogel precursor, the preparation of BMP2-hydrogel, and *in vitro* characterizations; B) The flowchart of the *in vivo* experimental design and characterizations of the BMP2-hydrogel treatment on the porcine model of LCPD.

In this study, we hypothesize that by first applying the bone wash technique and then injecting a BMP2-loaded hydrogel, we can achieve a broad distribution of the bioactive BMP2 within the necrotic head, further producing an advanced bone regeneration in the context of ONFH. To test the hypothesis, we synthesized a gelatin-heparin-tyramine (GHT) hydrogel as the BMP2 carrier. ^32^ We examined the leakage following the injection of the hydrogel and saline via optical observations after tissue clearing. We investigated the distribution of the injected hydrogel and saline via loading radiocontrast and micro-CT (mCT) scanning. The release profile of the BMP2-loaded hydrogel was examined over five weeks *in vitro*. The osteogenic bioactivity of the released BMP2 was tested *in vitro* by assays of qPCR and Alizarin red. (Fig. 1A) After that, we systemically assessed the *in vivo* effects of the BMP2-hydrogel treatment using a piglet model of LCPD. Visual, radiographic, histologic, histomorphometric, and mCT assessments were performed. (Fig. 1B)

## Results

### In vitro release of BMP2 from the BMP2-hydrogel

**Figure 2:**
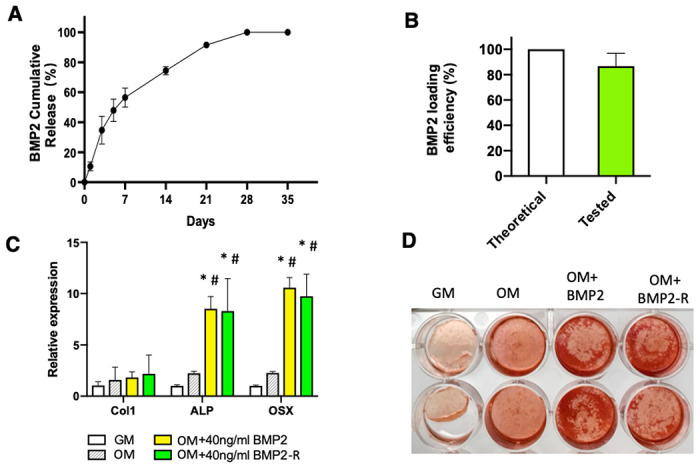
The GHT hydrogel can retain and preserve long-term bioactivities of BMP2. A) The graph showing the cumulative release profile of BMP2 from GHT hydrogel (0.75mg BMP2 per ml of hydrogel); B) The bar graph showing the total amount of detected BMP2 from the GHT hydrogel; C) The bar graph showing the relative gene expression by pig bone marrow mesenchymal cells (pBMSCs) cultured for 5 days in growth medium (GM), osteogenic medium (OM), OM supplemented with BMP2 (40ng/ml), or OM supplemented with released BMP2 (40ng/ml) from the hydrogel (BMP2-R); D) Alizarin Red staining of the pBMSCs that were cultured for 11 days in GM, OM, OM+BMP2(40ng/ml), and OM+BMP2-R(40ng/ml).

The BMP2 release, loading and release efficiency, and the bioactivity of the released BMP2 were tested *in vitro*. A high dose of BMP2 (0.75mg/ml) was mixed with hydrogel precursor, H_2_O_2_, and horseradish peroxidase (HRP) for the BMP2-hydrogel preparation. As depicted in Fig. 2A, the system only exhibited a 10% burst release of BMP2 in the first 24 hours. Near 60% of BMP2 was released over the first week. During the second and third weeks, a near-linear release profile was observed. More than 97% of BMP2 was released by the end of the fourth week. Five weeks after *in vitro* release, 88% of the theoretically loaded BMP2 was detected by an ELISA kit. (Fig. 2B) These data demonstrate that the GHT hydrogel can sustain BMP2 for four weeks, and the efficiency of BMP2 loading and release is high.

**Figure 3:**
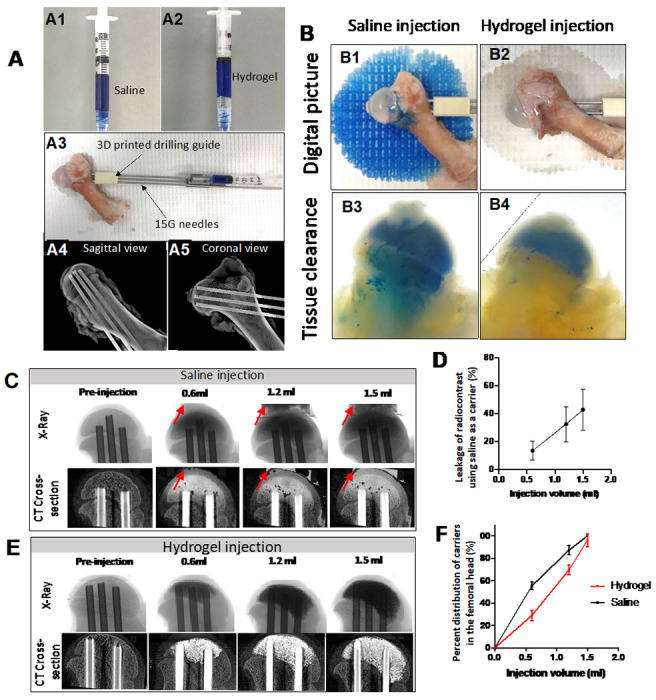
Injection of the hydrogel carrier produces a broad distribution in the femoral head without leakage. A) The pictures show the preparation for testing leakage *ex vivo*. A1 is the blue dye-labeled saline, and A2 is the blue dye-labeled hydrogel. A3 is the apparatus setup for testing the injection of hydrogel *ex vivo*, including the cadaveric porcine femur, 3D printed guide, and three drilling needles; A4 and A5 are X-ray images depicting the sagittal and coronal views post-drilling. B) The digital pictures show the leakage after the saline injection but not the hydrogel injection. B1 shows the blue dye-labeled saline leaking out from the porcine femoral head after injection; B2 shows no hydrogel leaking out from the porcine femoral head after injection; B3 & B4 are the images from tissue-cleared samples; B3 shows the backflow of saline to the metaphysis, whereas B4 shows no backflow of hydrogel to the metaphysis. C) Representative X-ray and 2D mCT images demonstrate the leakage that occurred when 0.6ml, 1.2ml, and 1.5ml of saline was injected (the red arrows show the leaked saline), and D) The graph shows the percentage leakage of saline; E) X-ray and 2D mCT images show no leakage when 0.6ml, 1.2ml, and 1.5ml of hydrogel was injected; F) The graph shows the distribution of saline and hydrogel in the femoral head after injection.

In order to test the bioactivity of the released BMP2 (BMP2-R), osteogenic assays were performed using pig bone marrow mesenchymal cells (pBMMC). The cells were cultured for five days. The qPCR data (Fig. 2C) showed that the pBMSCs exhibited an upregulation in gene expression for Collagen type 1 (Col 1), alkaline phosphate (ALP) and Osteorix (OSX) in the BMP2-R group compared to the cells cultured in osteogenic medium (OM, P < 0.0001) and growth medium (GM, P < 0.0001). The representative images of Alizarin red staining showed that the pBMMCs produced increased minerals after eleven days of culture in the BMP2-R group than the OM group (Fig. 2D). No significant differences were observed in the osteogenesis of pBMMC between BMP2-R and BMP2 groups (Fig. 3C & D). These data indicate that BMP2 released from the GHT hydrogel maintained its bioactivity.

### Ex Vitro injection of GHT hydrogel

The leakage of carriers was tested *ex vivo* using cadaveric femoral heads. The experimental apparatus setup is illustrated in Fig. 3A. The saline and hydrogel were both loaded with blue dye (Fig. 3A1 & A2) or a radiocontrast agent to visualize the leakage optically or by X-ray. A three-dimensional (3D) printed drilling guide was used for standardized drilling (Fig. 3A3). Before the injection, three intraosseous needles were placed and confirmed by the X-ray (Fig. 3A4 & A5). After a bone wash procedure, 1.5 ml of blue dye-loaded saline was injected. Figure 3B1 displayed that an abundant amount of blue dye leaked out and stained the paper. In contrast, only residual saline leaked out after the hydrogel injection (colorless wetting on the paper), and no blue dye leakage was observed (Fig. 3B2). The specimens were further processed with gradient alcohol for dehydration, followed by incubation in a refractive index matching solution consisting of benzyl benzoate and benzyl alcohol (BB/BA) for tissue clearance, which allowed for the visualization of blue dye inside the bone. Visual inspection following tissue clearance revealed that the blue dye was present in the metaphysis after saline injection and removal of the intraosseous needles, indicating a backflow of saline through the drill tunnels (Fig. 3B3). However, the blue dye was not present in the metaphysis tissue after hydrogel injection and removal of the needles, indicating the retention of the hydrogel in the epiphysis (Fig. 3B4).

The leakage and distribution of the two carriers were further quantified by mCT (Fig. 3C). Saline injections revealed leakage after the initial 0.6 ml injection, which increased with higher injection volumes. The leaked fluid was collected and the concentration of the radiocontrast was measured using X-ray intensities. The quantitation showed that over 40% of the radiocontrast leaked out of the femoral head when injecting saline. (Fig. 3D). In comparison, no radiocontrast was detected in the leaked fluid after hydrogel injection (Fig. 3E). Both the saline and hydrogel injections showed a wide distribution within the femoral head (Fig. 3F). The saline injection produced a wider distribution at a relatively low injection volume compared to the hydrogel injection. The results of these experiments suggest that using saline as the carrier can result in severe leakage of the loaded makers, but hydrogel is capable of retaining them within the femoral head.

### Visual and radiographic assessments of BMP2-hydrogel treatment on a piglet model of LCPD

**Figure 4:**
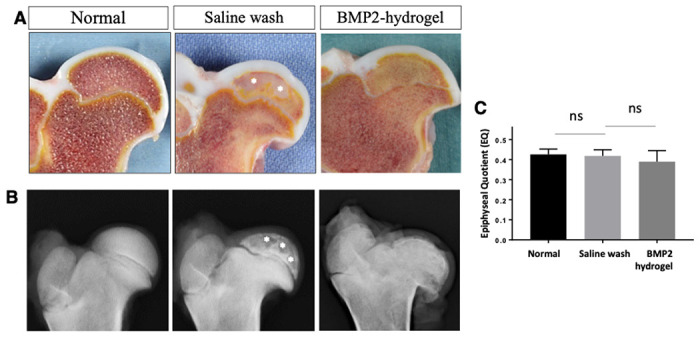
BMP2 hydrogel treated femoral head produced a semi-spherical shape with no bone defect A) Representative pictures of the bisected femoral heads from normal, saline wash, and BMP2-hydrogel treatment groups; B) Representative X-ray images of the femoral heads from the normal, saline wash, and BMP2-hydrogel treatment groups; C) Bar graph showing the mean EQ value of the femoral heads from the normal, saline wash, and BMP2-hydrogel groups. White asterisks indicate areas with large bone voids.

To investigate the treatment effects *in vivo*, the BMP2-hydrogel was locally administrated following a bone wash using a minimally invasive percutaneous technique on a piglet model of LCPD, as shown in Fig. 1. After an eight-week period following osteonecrosis induction, the piglets were euthanized, and the femoral heads were retrieved and bisected. As shown in Fig. 4A, the normal femoral head bone marrow appeared brownish, whereas a large region of the bone marrow in the saline wash group appeared whitish. The femoral head bone marrow of the BMP2-hydrogel group appeared brownish in the subchondral region and yellowish in the central region of the epiphysis. On X-ray, bone voids and a discontinued subchondral bone were present in the epiphysis of the saline wash group (Fig. 4B), whereas the epiphysis in the BMP2-hydrogel group had an intact subchondral bone similar to the control group and no bone voids. The deformity of the femoral head was used to evaluate the measurement of the epiphyseal quotient (EQ, maximum femoral head height/ maximum femoral head width). The mean value of EQ revealed no significant difference among the three groups (p = 0.26, ANOVA) (Fig. 4C).

The hip joint capsule and soft tissues were dissected to evaluate the occurrence of HO. Based on visual inspection and X-ray imaging, HO was not observed in the animals that received the BMP2-hydrogel treatment (Supplementary Information Fig. S1). Our previous study found ectopic bones in the hip capsule and soft tissue of animals that received the BMP2 saline treatment. ^21^

Taken together, visual and radiographic assessments indicate that the BMP2-hydrogel treatment produced a semi-spherical femoral head with a uniform bone matrix, while completely avoiding heterotopic bone formation.

### Histological assessments of bone regeneration and remodeling after BMP2-hydrogel treatment

**Figure 5:**
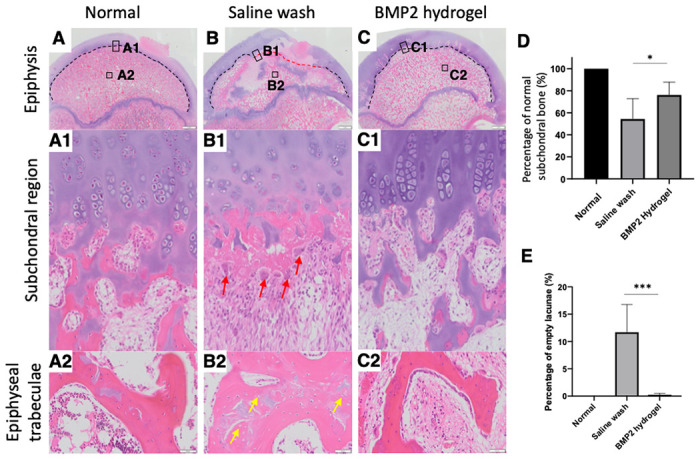
BMP2 hydrogel treatment repaired the subchondral and trabecular bones of femoral head A-C) Representative H&E staining images of the whole femoral heads of normal, saline wash, and BMP2-hydrogel groups; A1-C1) Magnified images of the subchondral regions of normal, saline wash, and BMP2-hydrogel groups; A2-C2) Magnified images of the epiphyseal trabeculae of normal, saline wash, and BMP2-hydrogel groups; D) Percentage of the subchondral region with restored endochondral ossification from the normal, saline wash, and BMP2-hydrogel groups; E) Percentage of empty lacunae from the normal, saline wash, and BMP2-hydrogel groups. Black dash lines show the normal subchondral bone. Red dash lines show the abnormal subchondral bone; Red arrows show the osteoclasts; Yellow arrows show the empty lacunae. * represent p <0.05; *** represent p <0.001.

Histological changes of the necrotic femoral head following the different treatments were visualized using H&E staining. Endochondral ossification (EO) was active in the subchondral region of the femoral head in the normal group, with visible hypertrophic chondrocytes columns and active endochondral bone formation (Fig. 5A, black dash line & Fig. 5A1). ONFH halted EO at the subchondral region (Fig. 5B red dash line & Fig. 5B1), with active osteoclastic resorption of the necrotic trabeculae (Fig. 5B1 red arrows) and partial restoration of EO in the subchondral region overtime (Fig. 5B, black dash line). In contrast, the BMP2-hydrogel treatment restored most of the subchondral EO, as shown in Fig. 5C1. 76 ± 11 % of the subchondral region showed restoration of EO in the BMP2-hydrogel group, compared with 54 ± 17% in the saline wash group (p = 0.02, Fig. 5D). The appearance of abundant empty lacunae is the hallmark of osteonecrosis. ^34^ In the saline wash group, a considerable number of empty lacunae were present (yellow arrows) in the epiphyseal trabeculae. However, a significantly decreased number of empty lacunae were found in the BMP2-hydrogel treatment group (Fig. 5E); 11.7 ± 4.5% of osteocyte lacunae were empty in the saline wash group versus 0.2 ± 0.2% in the BMP2-hydrogel group (p = 0.0003).

**Figure 6:**
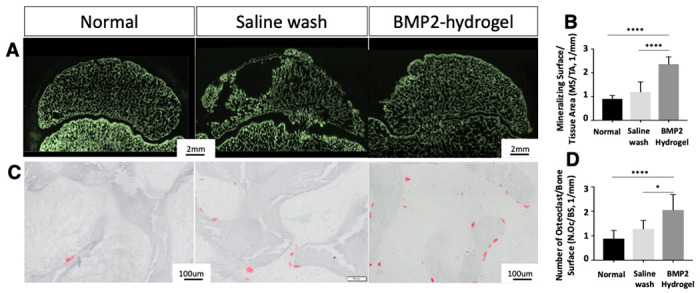
BMP2 hydrogel treatment accelerated epiphyseal bone regeneration and remodeling of femoral head A) Representative fluorescent microscopic images show the calcein (green) labeled bone surface in the femoral heads of the normal, saline wash, and BMP2-hydrogel groups; (B) Ratio of mineralizing surface to the total tissue area (MS/TA, 1/mm); C) Representative images of tartrate-resistant acid phosphatase (TRAP) staining show osteoclasts on the trabeculae in the normal, saline wash, and BMP2-hydrogel groups; D) Number of osteoclasts per bone surface (N.Oc/BS, 1/mm) of the normal, wash, and BMP2-hydrogel groups. * represents p <0.05; **** represents p <0.0001.

Undecalcified sections with calcine (green) labeling were used to evaluate the mineralizing surface (Fig. 6A). The mineralizing surface per total tissue area (MS/TA) of the BMP2-hydrogel group was significantly increased compared to the saline wash group (2.4 ± 0.3/mm vs. 1.2 ± 0.4/mm, p < 0.0001) and the normal group (0.90 ± 0.1/mm, p < 0.0001) (Fig. 6B). TRAP-stained sections were used to visualize osteoclasts on the epiphyseal trabeculae (Fig. 6C). The number of osteoclasts was significantly higher in the BMP2-hydrogel group compared to the saline wash group (2.2 ± 0.6/mm, vs. 1.6 ± 0.3/mm, p = 0.0486) and the normal group (0.7 ± 0.1/mm, p < 0.0001). However, the osteoclast number in the BMP2-hydrogel and saline wash groups were significantly higher than the normal group (Fig. 6D). The histological assessments indicate that BMP2-hydrogel accelerated epiphyseal bone regeneration and remodeling following ONFH. It largely restored EO at the subchondral regions, increased trabecular bone formation, and increased bone remodeling as noted by decreased empty lacunae and increased osteoclast number. As a result, most of the necrotic bone in the BMP2-hydrogel group was replaced by new bone.

### Assessing the epiphyseal bone architecture parameters of the BMP2-hydrogel treatment

**Figure 7:**
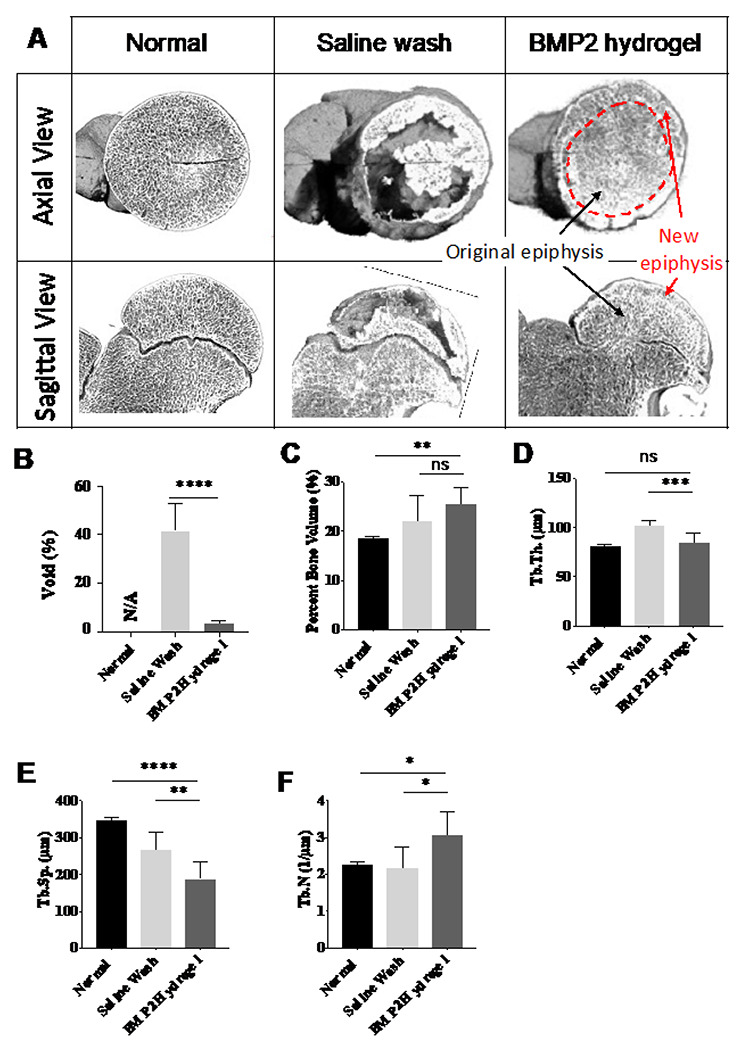
BMP2 hydrogel treatment produced a homogeneous bone regeneration of femoral head A) Representative 3D m-CT images of femoral heads from the normal, saline wash, and BMP2-hydrogel groups. Bar graphs showing B) the percentage of bone voids, C) the percentage of bone volume, D) the trabecular thickness (Tb.Th), F) the trabecular separation (Tb.Sp), and E) the trabecular number (Tb.N) of the normal, saline wash, and BMP2-hydrogel groups. * represents p <0.05; ** represents p <0.01; *** represents p <0.001; **** represents p <0.0001.

mCT was used to evaluate the parameters of the epiphyseal architecture of the femoral head after treatment. Typical 3D images from the three groups are shown in Fig. 7A. Both axial and sagittal views revealed a homogenous presence of trabecular bone in the normal and BMP2-hydrogel treated femoral heads. In the BMP2-hydrogel group, a visible addition of new bone (red arrow) was observed around the original epiphysis (black arrow), which confirms the restoration of EO in the subchondral region. In contrast, the femoral head of the saline wash group showed discontinuous new bone in the subchondral region with large bone voids throughout the epiphysis.

Histomorphometric measurements showed that 41% of the epiphyseal bone void was detected in the saline wash group, which is greater by eleven-fold than the BMP2-hydrogel treated group (3%, p < 0.0001) (Fig. 7B). The bone volume per tissue volume (BV/TV) of the BMP2-hydrogel group (25.6 ± 0.3%) was similar to the saline wash group (22.1 ± 4.6%, p = 0.22), but it was significantly higher than the normal group (18.5 ± 0.4%, p = 0.0085) (Fig. 7C). Mean trabecular thickness (Tb.Th.) was significantly decreased in the BMP2-hydrogel group compared to the saline wash group (84.3 ± 9.9 vs. 102.5 ± 4.9μm, p = 0.0005), but no significant difference was found between the BMP2-hydrogel and normal groups (81.7 ± 0.9μm, p = 0.77, Fig. 7D). Mean trabecular separation (Tb.Sp.) was significantly decreased in the BMP2-hydrogel group (188 ± 48pm) compared to the saline wash group (267 ± 47μm, P = 0.0084) and the normal group (347 ±10 μm, P < 0.0001) (Fig. 7E). Mean trabecular number (Tb.N.) was significantly increased in the BMP2-hydrogel group (3.1 ± 0.6/mm) compared to the saline wash group (2.2 ± 0.6/mm, p = 0.015) and the normal group (2.3 ± 0.6/mm, p = 0.026). (Fig. 7F). Taken together, this data suggest that the BMP2-hydrogel treatment achieved homogeneous bone regeneration.

### The effect of BMP2-hydrogel treatment on the growth plate

**Figure 8:**
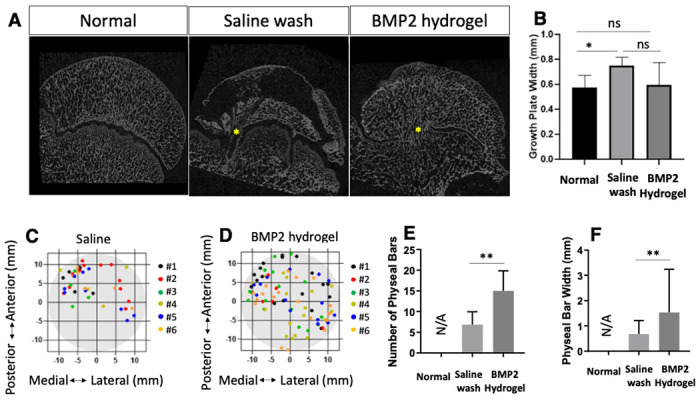
BMP2 hydrogel treatment increased the growth plate fusions. A) Representative 2D mCT images of femoral heads from the normal, saline wash, and BMP2-hydrogel groups. Yellow asterisks indicate areas of growth plate fusion; B) Bar graph shows the average growth plate width in the control, saline wash, and BMP2-hydrogel groups; C, D) Diagrams show the distribution of growth plate fusions after the (C) saline wash and (D) BMP2-hydrogel treatments; E, F) Bar graphs show (E) the average number of growth plate fusions and (F) the average width of growth plate fusions in different groups.

mCT was used to evaluate the treatment effect on the proximal femoral growth plate. As shown in Fig. 8A, the representative 2D mCT images exhibited areas of growth plate fusion in both the saline wash and BMP2-hydrogel treatment groups. No growth plate fusions were found in the normal group. The width of the growth plate in the BMP2-hydrogel treatment (0.60 ± 0.16 mm) was similar to the width of the growth plate in the normal group (p = 0.91). However, the width of the growth plate was significantly thicker in the saline treatment group (0.75 ± 0.06 mm) than the normal group (0.57 ± 0.09 mm, p = 0.016). (Fig. 8B). Figure 8C&D illustrate the distribution of the growth plate fusion in the saline wash (Fig. 8C) and BMP2-hydrogel (Fig. 8D) groups. Growth plate fusion occurred more frequently in the anterolateral region. The average width and number of growth plate fusions were 1.5 ± 0.2 mm and 15 ± 2/head in the BMP2-hydrogel group respectively, which is a significant increase compared to the saline wash group (0.7 ± 0.1 mm, p = 0.0022, and 7 ± 1/head p = 0.0061) (Fig. 8E & F). This result indicates that both saline wash and BMP2-hydrogel treatments can affect the proximal femoral growth plate, but the BMP2-hydrogel treatment exhibited a significantly higher number and width of growth plate fusions.

## Discussion

LCPD is a severe pediatric bone disease that can lead to disabling osteoarthritis. Local delivery of biological agents offers potential new treatment options.^20^ However, there is a lack of effective delivery methods which would provide a broad and sufficient local osteoinduction for homogenous bone regeneration. To address this clinical need, we developed a BMP2-hydrogel treatment via a transphyseal bone wash and subsequential injection of BMP2-loaded hydrogel. We found that the new BMP2 delivery strategy can provide broad BMP2 distribution within the necrotic head with no leakage during the injection, thereby restricting the loaded BMP2 within the target region for local osteogenic induction. The GHT hydrogel can retain the bioactive BMP2 for four weeks *in vitro*. The *in vivo* experiments using a piglet model of LCPD showed that the BMP2-hydrogel treatment significantly increased the restoration of endochondral ossification at the subchondral region, and produced a near-complete healing of the epiphyseal bone while preventing HO. While performing the *in vivo* experiments, we discovered that the BMP2-hydrogel treatment increases the number and width of the growth plate fusions. These findings demonstrate the potent effects of local BMP2-hydrogel treatment on bone regeneration following ischemic osteonecrosis and its concerning effect on the growth plate. Given these findings, local BMP2-hydrogel treatment may be promising for teenagers and young adults whose growth plates are not active. Further studies are warranted to optimize the delivery of local BMP2-hydrogel and the use of transphyseal drilling technique in juvenile femoral heads.

### Bone wash reconditions the local necrotic microenvironment and facilitates the distribution of biomaterial.

The harsh necrotic microenvironment is one of the major challenges for bone repair following ONFH. ONFH produces and leaves an abundance of necrotic debris and pro-inflammatory factors in the bone marrow space, including necrotic fat and debris as well as damage-associated molecular patterns. The necrotic debris activates and sustains local innate immune responses, leading to chronic inflammation, increased bone resorption, and decreased bone formation.^35,36^ To improve the local microenvironment, traditional core decompression procedures are used to remove a large piece of necrotic bone (8-10mm). However, the procedure raises concerns for iatrogenic complications such as subtrochanteric fracture, inadvertent penetration, or collapse of the femoral head.^37^ Here, we applied three epiphyseal drillings and followed with an intraosseous bone wash, which minimized the disruption of the native trabecular network by using small drillings (≤3mm). It has been reported that multiple epiphyseal drillings (MED) could produce multiple dispersive tunnels to the necrotic bone, which may be more effective for vascular restoration than one large tunnel.^11^ More importantly, MED drilling tunnels can be used as inflow and outflow portals for the intraosseous saline wash. Our previous study reported that the bone wash following the MED can significantly remove debris in the necrotic bone marrow space such as fats, DNA fragments, and pro-inflammatory proteins.^29,33^ As a result, the washed epiphyseal bone provides a “porous scaffold” facilitating new tissue ingrowth and angiogenesis. Our previous study also revealed that the bone wash process could significantly improve bone regeneration following ONFH, as compared with the MED drillings or with no treatment on the piglet LCPD model.^33^ (new analysis of the bone wash group also presented in this study, Fig. 4–7). Yet, MED and bone wash procedures could not produce complete regeneration of the necrotic epiphysis, and large bone voids were observed within the femoral head (Fig. 4–7). Moreover, nearly half of the subchondral region exhibited halted endochondral ossification (Fig. 5). With incomplete healing and restoration of endochondral ossification, there is a high risk of development and progression of femoral head deformity.^33^

### GHT hydrogel provides an ideal carrier for local BMP2 administration in the ONFH treatment.

Local delivery of BMP2 can dramatically improve osteoinduction in the treatment of ONFH. However, the major concerns of BMP2 are HO and long-term bioactivities. A high dose of BMP2 is commonly needed for long-lasting osteoinduction, which further increases the risk of HO. A prospective clinical study reported a more frequent occurrence of HO in patients receiving BMP2 (4mg per hip, 8/66 hips) than those not receiving BMP2 (1/75 hips).^38^ In our previous study, local BMP2 administration (1mg BMP2 per hip) exhibited a high incidence of HO when using saline as a carrier (4/6 hips). ^21^ Based on our previous studies, we selected the GHT hydrogel, which has a heparin content of 7.7% (w/w). ^30,32^ The GHT hydrogel not only retained BMP2 for four weeks but also preserved the high pro-osteogenic activity of the released BMP2 (Fig. 2).

In addition to a well-designed controlled release, the physical features of the GHT hydrogel play an important role in maintaining a high local osteoinduction and preventing HO formation. The injectability of the GHT hydrogel system can be easily adjusted by changing the precursor concentration. ^32^ 2% GHT hydrogel has a modest injectability. Compared to a saline carrier, 2% GHT hydrogel has less flowability, which can be injected, spread, and confined within the trabecular space (Fig. 3). When injecting the hydrogel, a conservative injection volume is recommended to avoid leakage by overdose. The injection volume should be determined according to the size of the femoral head. For an eight to nine weeks old male Yorkshire piglet, the total marrow space of the femoral head ranged from 1.8ml - 2ml. Therefore, 1,5ml of the hydrogel was applied for both *in vivo* and *ex vivo* experiments, and no leakage was observed.

As a result of a well-controlled BMP2 release and appropriate injectability, the BMP2-hydrogel treatment produced robust osteoinduction within the necrotic head. After seven weeks of treatment, the BMP2-hydrogel treated femoral heads exhibited homogenous bone regeneration (Fig. 5). The epiphyseal bone showed a high level of bone formation and remodeling, which was reflected by a low ratio of empty lacunae (Fig. 5E) and increased bone formation (MS/TA, Fig. 6B) and bone resorption (N.Oc/TA, Fig. 6D). We also observed a high ratio of restored endochondral ossification in the subchondral region (Fig. 5D). Compared to the previous study which used saline for BMP2 delivery, we found less femoral head deformity, more homogeneous bone regeneration, and avoided HO in the current study. ^21^ Therefore, the use of GHT hydrogel for the local delivery of BMP2 provided a broad and effective bone regeneration for ONFH.

### Limitations and outlook of the BMP2-hydrogel treatment for ONFH

The study does have limitations that may warrant further investigation into the application of the BMP2-hydrogel treatment. First, the BMP2-hydrogel treatment can cause growth plate fusion. Both mCT and histology revealed small osseous bridges between the epiphysis and the metaphysis through the growth plate. It is reported that cross-growth plate drillings using large needles can damage the growth plate and lead to growth disturbance. ^39–41^ Makela et. al, reported that small needle drillings would not affect limb growth as long as the affected growth plate area was lower than 7%. The current study applied three 15G (1.8mm) needles which represent 2.4% of the GP area. Significantly increased growth plate fusions were observed following the treatments. However, we were not able to evaluate how it affected limb growth as an above-knee amputation was performed, which precluded accurate measurement of the femoral length Further investigation is necessary to confirm the influence of the BMP2-hydrogel treatment on femur growth. We do believe that transphyseal drilling with the application of BMP2-hydrogel treatment should be limited to teenagers or young adults with less active or inactive growth plates.

Second, the study only considered the early stage of ONFH. Our findings cannot represent the full range of clinical situations, such as later stages with epiphyseal collapse and deformity. We studied the early, pre-collapse stage as it is the ideal time to institute a femoral head preservation treatment. Based on the excellent bone regeneration observed, we can foresee that the BMP2-hydrogel treatment may also improve outcomes for later stages of ONFH when combined with procedures to improve the femoral head deformity and provide mechanical support using bone graft or bone substitute. ^42,43^

Third, the safety of this treatment needs further investigation prior to clinical translation. The pig model used in this investigation is a severe model that produces complete ischemic osteonecrosis of the femoral head. Therefore, the bone wash and hydrogel delivery can be performed with no consideration for potential damage to the normal bone marrow tissue. In some patients, however, there may only be partial osteonecrosis of the femoral head, and the necrotic bone and normal bone may be in close proximity. In such cases, a rigorous bone wash may disrupt the normal bone marrow tissue. Therefore, further studies are required to test the effect of the bone wash and BMP2-hydrogel injection on normal bone marrow tissue.

In summary, local BMP2-hydrogel injections after a bone wash procedure using a multi-needle technique produced homogeneous bone regeneration while preventing HO. The combined treatment of the BMP2-hydrogel and the bone wash technique may be a potential ONFH treatment for teenagers and young adults with inactive growth plates.

## Materials And Methods

### Materials

Gelatin (Type B, from bovine skin, 225 g Bloom, average molecular weight = 50 kDa, Cat# G9391), heparin (sodium salt from porcine intestinal mucosa, MW =17–19 kDa), Type I collagenase (from Clostridium histolyticum), calcein (Ex/Em 495/517nm, cat.no.C0875) were purchased from Sigma Aldrich (St Louis, MO, USA). The gelatin-tyramine-heparin hydrogel precursor (GTH) was synthesized as previously reported.^32^ Horseradish peroxidase (HRP, 304 units/mg) was purchased from Thermo Scientific (Rockford, IL, USA). Hydrogen peroxide (H_2_O_2_) aqueous solution (35%, w/w) was purchased from BDH Chemicals (Westchester, PA, USA). The radio-contrasts, iopamidol (Isovue) and barium sulfate (BaSO_4_) suspension were purchased from Bracco (Milan, Italy). The recombinant human BMP2 powder was obtained from INFUSE^®^ Bone Graft (Medtronic, Minneapolis, MN) and reconstituted in sterile water according to instructions from manufacture. BMP2 Quantikine ELISA Kits were purchased from R&D Systems, Inc. (Minneapolis, MN, USA).

### Preparation of GTH hydrogel and BMP2-hydrogel

The solution of GTH hydrogel precursors was prepared by dissolving GTH hydrogel precursors into phosphate-buffered saline (PBS, Gibco). For the ex vivo study, GTH hydrogel was prepared by mixing 1.5ml of the GTH solution (2%, w/v), 7.5ul of HRP solution (200unit/ml), 7.5 ul of peroxide hydrogen (0.4mM), and one drop of blue dye (Salis International, Inc., Oceanside, CA) in a 3ml syringe. The mixture was gently shaken by hand, and then left standing for 2mins.

For in vivo studies and release kinetics, 1.5 ml of BMP2-hydrogel was prepared by mixing 0.75 ml of BMP2 solution (1.34 mg/ml), 0.75 ml of GTH solution (4%, w/v), 7.5ml of HRP solution (200unit/ml) and ml of peroxide hydrogen (0.4mM) in a 3ml syringe with cap. Before mixing, all the solutions were sterilized by passing through the 0.22 mm filter (Corning^™^ Disposable Vacuum Filter). After 2mins setting at room temperature, the hydrogel was stored at 4°C until injection. The concentration of BMP-2 chosen for this study was based on our previous results.^21^

### In Vitro Release BMP-2 from the GTH Hydrogel

100ul of BMP2-hydrogel was injected through 24G needles into the insert of a 24-well transwell system. The insert and the BMP2-hydrogel were incubated in 1 ml PBS buffer at 37°C. At a specific time point, the release medium was collected and stored at −80°C with 1 ml fresh medium replaced. At the last collection, the gels were soaked in 1 ml release buffer containing 30 units/ml type I collagenase, and the BMP-2 in the final solution was defined as unreleased BMP-2. After the dissolution of the gel, all the samples were thawed and quantified using a BMP-2 Quantikine ELISA Kit (R&D systems). The initially bound BMP-2 was determined by adding all the released BMP-2 and unreleased BMP-2 together. Three samples were prepared at each time point, and each experiment was repeated twice.

### Ex Vivo Injection of GTH Hydrogel

Twelve femoral heads were collected from cadaveric pigs of six to eight weeks old. The specimens were collected under a clean condition in an operating room, wrapped in saline-soaked gauze, and stored at −20 °C. To mimic the epiphyseal osteonecrosis ex vivo, the samples were subjected to three thaw-freeze cycles. For each cycle, the samples were placed into a 37 °C water bath for 6 hours, followed by a −20 °C freezer for over 4 hours. After that, three 15-gauge intraosseous needles were placed within the femoral epiphysis using a transphyseal approach and a 3D-printed needle guide for parallel needle placement. The inter-needle distance was 8 mm. The needle placement was considered satisfactory based on X-ray imaging when the needles were in the mid-coronal plane of the epiphysis, crossing the physis by at least 2.5 mm. Then, an epiphyseal bone wash was performed to remove bone marrow debris. Two 30mL syringes were connected to two of three needles using a Luer-Lock. The three intraosseous needles were used alternatively as inflow and outflow portals for the saline wash. 30 mL of pre-warmed saline was used, and 16 washes were performed per sample with a total wash volume equaling 480 mL per sample. Finally, 1.5 ml of the hydrogel was injected into the epiphysis with 0.5ml for each needle. To visualize the leakage, we loaded blue dye or radiocontrast during the preparation of the hydrogel (n = 6). The injection of saline (with blue dye or radiocontrast) was used as the control (n = 6).

### Animals

The study was approved by the local Institutional Animal Care and Use Committee at the University of Texas Southwestern Medical Center (Protocol number: 2016-101442-USDA). A total of twelve male Yorkshire piglets aged between six to eight weeks (25 to 35 lbs.) were obtained from a breeder for the in vivo study (K-Bar Livestock, LLC, Sabinal, TX). All animals were maintained under environmental controls consistent with the Guide for the Care and Use of Laboratory Animals.

### Induction of Osteonecrosis and Local Administration of BMP2-Hydrogel

Ischemic osteonecrosis in the femoral head of a piglet was induced on the right limb as previously described. ^33^ One week following the induction, the piglets received three percutaneous epiphyseal drillings using three 15-gauge intraosseous needles, followed by the bone wash procedure using 480ml prewarmed saline. After the bone wash, 1.5ml of BMP2-hydrogel was injected through the three drilling needles (0.5ml per needle). All piglets received a local non-weight-bearing treatment via above-knee amputation using a previously described method on the right limb. ^33^ The non-weight-bearing treatment was instituted postoperatively to minimize femoral head deformity and to simulate the clinical practice of recommending local non-weight-bearing treatment postoperatively in patients with active stage of LCPD.

### μCT and Morphometric Assessment

Following euthanasia, the femoral heads were bisected coronally and fixed in 10% neutral buffered formalin. After fixation, all femoral heads were scanned using a μCT (Skyscan 1172, Bruker-μCT, Kontich, Belgium) at a setting of 100 kV and 100 μA and a resolution of 13.3 μm/pixel as previously described ^33^ and reconstructed with NRecon (version 1.7.0.4; Bruker-μCT, Kontich, Belgium). The reconstructed images were binarized to a common threshold using CTAn (version 1.13.5.1; Bruker μCT), where the region of interest was defined to capture the original necrotic epiphysis. The region of interest was outlined within the epiphysis, avoiding the subchondral region of the calcified epiphyseal cartilage. The CTAn software was used to calculate the three-dimensional morphometric values for percent bone volume, trabecular thickness, trabecular number, trabecular separation, and percent bone void volume in the epiphysis of the femoral heads. To determine a bone void volume, spaces with a trabecular separation larger than 570 microns were defined as bone voids, as the control group had a maximum trabecular separation of 570 microns.

### μCT Assessment of Epiphyseal Quotient

Anteroposterior X-ray images were used to measure the epiphyseal quotient that reflects the amount of femoral head collapse. The epiphyseal quotient is defined as the maximum height divided by the maximum width of the femoral head. ^44^

### Histology, bone histomorphometry, and fluorochrome labeling Analysis

The bisected femoral head specimens were dehydrated in a series of graded ethanol solutions after μCT scanning. The anterior half of the femoral head was processed in methyl methacrylate (MMA) for plastic embedding, whereas the posterior half of the femoral head was decalcified in ethylenediaminetetraacetic acid (EDTA) and embedded in paraffin. Both portions were sectioned at a thickness of 4 μm.

The paraffin sections were used to perform hematoxylin and eosin (H&E) staining using a standard protocol.^45^ The percentage of the restored subchondral endochondral ossification was evaluated using H&E stained images by measuring the length of the osteochondral junction with new bone formation, normalizing the value with the total length of the osteochondral junction. The empty osteocyte lacunae were counted, as defined by osteocyte lacunae with an absence of the cell body or lacunae containing only a pyknotic nucleus.^46^

The plastic sections were stained with tartrate-resistant acid phosphatase (TRAP) to determine the number of osteoclasts. ^33^ TRAP-positive cells were counted and normalized to bone surface (N/BS). Plastic sections were also imaged to determine the percentage of mineralizing surface via quantifying the fluorochrome-labeled bone surface per tissue area (MS/TA). All sections were imaged using the OSTEOIMAGER Scanner and analyzed using the BIOQUANT OSTEO software.

### Statistical Analysis

For the epiphyseal quotient, μCT morphometric, histologic, and histomorphometric measurements, a one-way analysis of variance (ANOVA) was performed to determine the overall difference among the 3 groups. If the difference was significant (p < 0.05), a post-hoc Tukey honestly significant difference test was performed to assess the significance among groups.

## Data Availability

The datasets used and/or analyzed during the current study are available from the corresponding author upon reasonable request.
